# The relative meaning of absolute numbers: the case of pain intensity scores as decision support systems for pain management of patients with dementia

**DOI:** 10.1186/s12911-015-0233-8

**Published:** 2015-12-24

**Authors:** Valentina Lichtner, Dawn Dowding, S. José Closs

**Affiliations:** School of Healthcare, University of Leeds, Leeds, UK; Columbia University School of Nursing, New York, NY USA; Center for Home Care Policy and Research, Visiting Nurse Service of New York, New York, NY USA

**Keywords:** Pain measurement, Dementia, Aged, Hospitalization, Decision making, Clinical decision support systems, Qualitative research

## Abstract

**Background:**

Assessment and management of pain in patients with dementia is known to be challenging, due to patients’ cognitive and/or communication difficulties. In the UK, pain in hospital is managed through regular assessments, with the use of pain intensity scores as triggers for action. The aim of this study was to understand current pain assessment practices, in order to later inform the development of a decision support tool designed to improve the management of pain for people with dementia in hospital.

**Methods:**

An exploratory study was conducted in four hospitals in the UK (11 wards), with observations of patients with dementia (*n* = 31), interviews of staff (*n* = 52) and patients’ family members (*n* = 4) and documentary analysis. A thematic analysis was carried out, structured along dimensions of decision making. This paper focuses on the emergent themes related to the use of assessment tools and pain intensity scores.

**Results:**

A variety of tools were used to record pain intensity, usually with numerical scales. None of the tools in actual use had been specifically designed for patients with cognitive impairment. With patients with more severe dementia, the patient’s body language and other cues were studied to infer pain intensity and then a score entered on behalf of the patient. Information regarding the temporality of pain and changes in pain experience (rather than a score at a single point in time) seemed to be most useful to the assessment of pain.

**Conclusions:**

Given the inherent uncertainty of the meaning of pain scores for patients with dementia, numerical scales were used with caution. Numerical scores triggered action but their meaning was relative - to the patient, to the clinician, to the time of recording and to the purpose of documenting. There are implications for use of data and computerized decision support systems design. Decision support interventions should include personalized alerting cut-off scores for individual patients, display pain scores over time and integrate professional narratives, mitigating uncertainties around single pain scores for patients with dementia.

**Electronic supplementary material:**

The online version of this article (doi:10.1186/s12911-015-0233-8) contains supplementary material, which is available to authorized users.

## Background

Since the mid 1990s pain has been accepted internationally as a fifth vital sign [1], with recommendations for its measurement and documentation to be included in medical and nursing guidance. There has long been recognition that the assessment and management of pain in patients in hospital has been poor [2–4], and is particularly challenging in patients with cognitive impairments such as dementia [5]. This is due in part to persons with dementia having difficulties in recalling earlier pain experiences, understanding that the current experience they are having is pain and expressing their responses to pain meaningfully, making communication of pain experiences problematic. They may lose semantic memory of pain as a concept [6] and may express and communicate their pain in unexpected ways, such as becoming agitated. Because of these difficulties clinicians find it challenging to recognize the presence of pain in people with dementia, as well as being able to assess its nature and intensity, hampering their ability to manage pain effectively. There are significant consequences related to the inadequate management of pain in hospital settings, including slower functional rehabilitation, increased depression, longer hospital stays and poorer quality of life [7].

In some contexts, such as in the UK, US and Australia, the traditional approach to ensuring pain is managed effectively is to ensure a system of regular pain assessment, with an assumption that this will lead to improved pain management. Systems of regular pain assessment are linked to the use of structured pain assessment tools, and in particular, numeric rating scales (NRS) [8]. Patients’ pain intensity scores may be gathered by asking patients to self-report with respect to the scale. The NRS is usually a 0 to 10 scale, with zero representing no pain and ten the worst possible pain. However, while numerical scales have demonstrated good validity and reliability in terms of their ability to represent pain intensity [9–11], they are not without their problems. For example: patients’ use of NRS may be “idiosyncratic and inconsistent” [12]; chronic pain patients may have a reduced number sense compared to those with acute pain [13]; and while patients who have early or moderate dementia may be able to use a NRS [14, 15], it is challenging to determine at what point the validity of self report is no longer adequate and different approaches to assessment are required. There has also been considerable debate about ‘cut-points’, i.e. which scores out of 10 represent mild, moderate or severe pain. Studies have been inconclusive, indicating high variability of these within samples of patients, making their use as standardized triggers for pain management interventions questionable [14, 16, 17] and inviting instead patient-specific triggering thresholds.

As an alternative to NRS, a large number of observational assessment tools have been designed for the assessment of pain in patients with dementia, informed by guidance published by the American Geriatrics Society [18]. Observed patient behaviour, facial expression, negative vocalisation, body language, changes in activity patterns, changes in interpersonal interactions, are used in these tools as a proxy for the presence or absence of pain. A large number of these tools also include some form of scoring of pain intensity. This may be done by counting checkmarks in a checklist (i.e. yes/no binary responses for item present or absent), or by reference to a variety of rating systems (Likert scales, binary scores, multiple choice and visual analogue scale systems), with large variability of scoring range (from 0–6 to 0–60). However, data on reliability, validity and clinical utility of these observational tools are limited [19], and there is some evidence to suggest that they are not routinely used in clinical practice.

Computerized decision support systems (CDSS) are one of the ways in which pain assessment and management could be better supported in hospital settings [20]. CDSS “provide clinicians with patient-specific assessments or recommendations to aid decision making” [21]. Their use has been shown to improve care processes and patient outcomes (e.g.[20]); however their successful implementation and use depends on ensuring that they fit with the organizational context and clinician workflow (e.g.[22]). CDSS therefore need to have clinical utility. The evaluation of the clinical utility of tools is multi-dimensional [23], including whether or not an intervention is clinically effective, whether it can provide an economically efficient solution to a problem and whether it is clinically useful (i.e. if the usefulness, benefits and drawbacks mean that it is adopted into work routines and practices).

In this paper we report findings from an exploratory study to investigate how pain assessment and management tools are currently used for assessing and managing pain in a sample of patients with dementia in acute hospital settings in the United Kingdom. The study is part of a larger programme of work aimed at developing decision support interventions to help with the assessment and management of pain in patients with dementia. The scope of this paper is limited to the use of pain intensity scores and staff perceptions on their clinical utility. We consider the implications of our findings for the design of CDSS for pain assessment and management.

## Methods

We used an embedded case study design [24], in four NHS hospital organisations in England and Scotland. Patients, wards and hospital organisations were each studied as cases. Ethnographic non-participant observations were centred on the patients - their experience of pain, their interaction with healthcare professionals and the care they received. The study design included a mix of qualitative research methods; observations of patients at bedside; observations of the context of care, including informal open conversations with patients, their family and ward staff; audits of patient hospital records; documentary analysis of artefacts; and semi-structured interviews with staff and families. Data were collected in a minimum of two wards in each hospital site. Data gathered through observations and conversations in the wards were recorded in field notes; interviews were audio-recorded (and transcribed) whenever participants gave their consent.

Observations were designed as ‘non-participant’ in the sense that field researchers did not take part in patient care activities. As it is often the case in ethnographic work, the researchers attempted to ‘become part of the furniture’ (e.g. [25]), by spending time in the settings they studied so that staff became familiar with them and carried on with their activities as if researchers were not there observing; the researchers attempted to minimise any impact their presence at bedside or in the ward may have on staff activities, or their use of tools, or staff interaction with the patient. On the other hand, having the researcher at bedside, did inevitably affect patients’ interaction with the environment and patients’ experience of their hospital stay, for example from being alone for long hours to being with some company (we reflected on this in a paper [26] written at the time of data collection).

### Sampling of settings

The 4 NHS hospital organisations included in the study were geographically located across various regions of the UK (the South East, North East, North West of England and Scotland). They were teaching hospitals, all located in large urban centres, and provided care to patients across their local region. Only 1 of the 4 hospitals had implemented a hospital-wide electronic health record (EHR) system, but at the time of the study regular nursing observations were still documented on paper charts. The 11 wards covered a variety of specialities including acute admissions, surgical wards (vascular and general surgery/orthopaedic), elderly medicine, rehabilitation and continuing care, sampled to gain insight into a wide range of clinical areas where patients with dementia may be. On average the wards operated on nurse to patient ratios of from 1 nurse to 8 patients up to 1 nurse to 14 patients.

### Sampling of participants

Researchers at the four sites invited inpatients to participate in the study, as long as they were over 65 years of age, had a recorded diagnosis of dementia and family members were available at the time of consent. All patients matching these inclusion criteria, who were staying in the wards at the time of the study, were invited to take part (however, often the diagnosis of dementia was not recorded in the patients’ records and although patients were known by clinicians in the ward to have dementia, these patients could not be recruited). Their family members (‘carers’) were also invited to participate in interviews.

All ward staff were invited to take part in interviews, as well as managers and specialists in hospital services relevant to the care of the patients participating in the study.

### Ethics, consent and permissions

Ethical approval was granted by Ethics Committees in England and Scotland (NRES Committee Yorkshire & The Humber - Leeds West - REC reference: 12/YH/0363; Scotland A Research Ethics Committee, Edinburgh - REC Reference 13/SS/0006). Following guidance of the Mental Capacity Act 2005 and the Mental Health (Care and Treatment) (Scotland) Act 2003, patients’ written consent was subject to capacity assessment, agreement from family members and consultation with staff. Written consent was also obtained from all members of staff and family members who participated in interviews. The researchers complied with local research governance requirements for data collection of the NHS organisations that agreed to participate in the study. Data were anonymised at the time of data collection.

### Data collection methods

Following patient recruitment and consent, the researcher sat at the patient’s bedside, observing, and whenever possible talked with the patient, about their pain, their experiences of the care they received, or anything else the patient wished to converse about. Some patients were too unwell and others with more severe dementia were too confused to engage in conversations, but an attempt was always made to relate to the patient, and understand their experience of pain (if any). Observations of the context of care were done both from the bedside and from communal areas of the ward (such as corridors and nursing stations). During this time, informal conversations with staff, families and visitors were also an opportunity to ask for clarifications of observations made and gather information about decision making processes, complementing data gathered through interviews. Each individual patient was observed across different time points in the day for up to 3 days. Researchers made extensive field notes, recording their observations of who interacted with the patient, conversations that were undertaken with them, and their behaviour. Researchers consulted patients’ hospital paper-based records, noting any reference to assessment or management of pain; they identified pain assessment tools (or related documentation) used on each ward and conducted semi-structured interviews with staff, including physicians, nurses and healthcare support workers, as well as family members of the patient. The interviews focused on the participants’ views and recollections of how pain was recognised, managed and documented in each of the wards, what pain assessment tools were used, if any, and what improvements could be made to practice (interview guides provided in Additional file 1). The majority of interviews (44 out of 56) were recorded and transcribed and field notes recorded for the remainder when participants did not consent to the audio-recording. Data were collected in the period May 2013 to July 2014.

### Data analysis

A thematic analysis of the data was carried out, using both inductive and deductive approaches. NVivo [27] was used to support the analytic process, with all data files being stored on a central SharePoint [28] space that could be accessed by all individuals in the research team. Data from interview transcripts were coded separately from those of observations and audits of patients’ records. Interviews generated themes more specifically related to decision making processes (e.g. information types, judgments, rationales for decisions) as participants explained their actions and thought processes; field notes from observations gave insight into the context of care, and what ‘actually happened’ in terms of patient care and patient-staff interaction; audit of patient medical and nursing notes provided evidence on the documentation of pain assessment and management. Data from the three sources were compared and integrated to provide a more nuanced understanding of events and processes. For example, how activities planned for given times actually took place at different times (or not at all), or how the documentation of assessment failed to describe the richness of the whole actual activity and staff interactions with the patient.

Team analysis meetings, attended by the researchers from each of the sites and investigators, were held monthly during and after the data collection period, and findings were compared and discussed within the group. The thematic analysis was structured along dimensions of decision making – including pain assessment, pain management and decision support – but a wide range of other different themes also emerged from the data. The use of pain intensity scores emerged as an important aspect of pain assessment and management practices in the hospitals studied; data related to this area of practice were subsequently extracted and subject to separate analysis, which is the focus of this paper. The first author (VL) extracted this subset of data, identified initial relevant themes (e.g. the subjectivity of pain intensity scores) and discussed them with the co-authors (SJC and DD). The resulting analysis was shared with the other researchers on the project who were asked to confirm the findings or discuss differences in interpretation. These were resolved by consensus and integrated into the analysis. Findings are illustrated below with quotes from interviews’ transcripts, chosen for their clarity and representativeness, but the subset of data for analysis was inclusive also of field notes from observations and audits of patient records.

## Results

Table 1 provides a summary of the data collected and patient characteristics. Patients who participated in the study were elderly with a mean age of 88 years (range 75-99) all with a diagnosis of dementia with varying degrees of cognitive impairment. We carried out a total of 170 h of direct observation spending more than 480 h in the field. We interviewed 52 members of staff, including 8 physicians, 33 among ward nurses, specialist nurses and ward managers, 6 healthcare assistants, 3 clinical educators, 1 physiotherapist, 1 pharmacist and a director of nursing. We interviewed family members (spouses or children) of 4 of the patients we observed, who consented to participate in recorded interviews. An unrecorded larger number of family members or visitors (some as elderly as the patients themselves) engaged in impromptu conversation during observations in the hospital wards.

**Table 1:** Types of data collected and participant characteristics

	H1	H2	H3	H4	Total
Patients Observed (N)	8	7	9	7	31
Interviews with staff (N)	24	13	7	8	52
Interviews with patients’ family members (N)	1	3	0	0	4
Total time spent observing patients (approx. hours)	71 h	45 h	22 h	32 h	170 h
Total time in the field (approx. hours)	161 h	167 h	73 h	85 h	486 h
Mean patient age (range)	83 (77–87)	84 (75–93)	88 (79–99)	85 (75–94)	88 (75–99)
Patient Gender	Male = 1 Female = 7	Male = 2 Female = 5	Male = 4 Female = 5	Male = 4 Female = 3	Male = 11 Female = 20

### Pain assessment practices and pain intensity scores

Different wards had different rules and routines in place and these influenced how pain was assessed and managed [29]. In surgical wards, for example, acute pain was expected for all patients and protocols were in place to prescribe analgesia on a regular basis; other wards, such as elderly care, expected patients with more of a mix of chronic and acute pain, and pain assessment and management were more ad-hoc on a patient-by-patient basis.

**Fig. 1:**
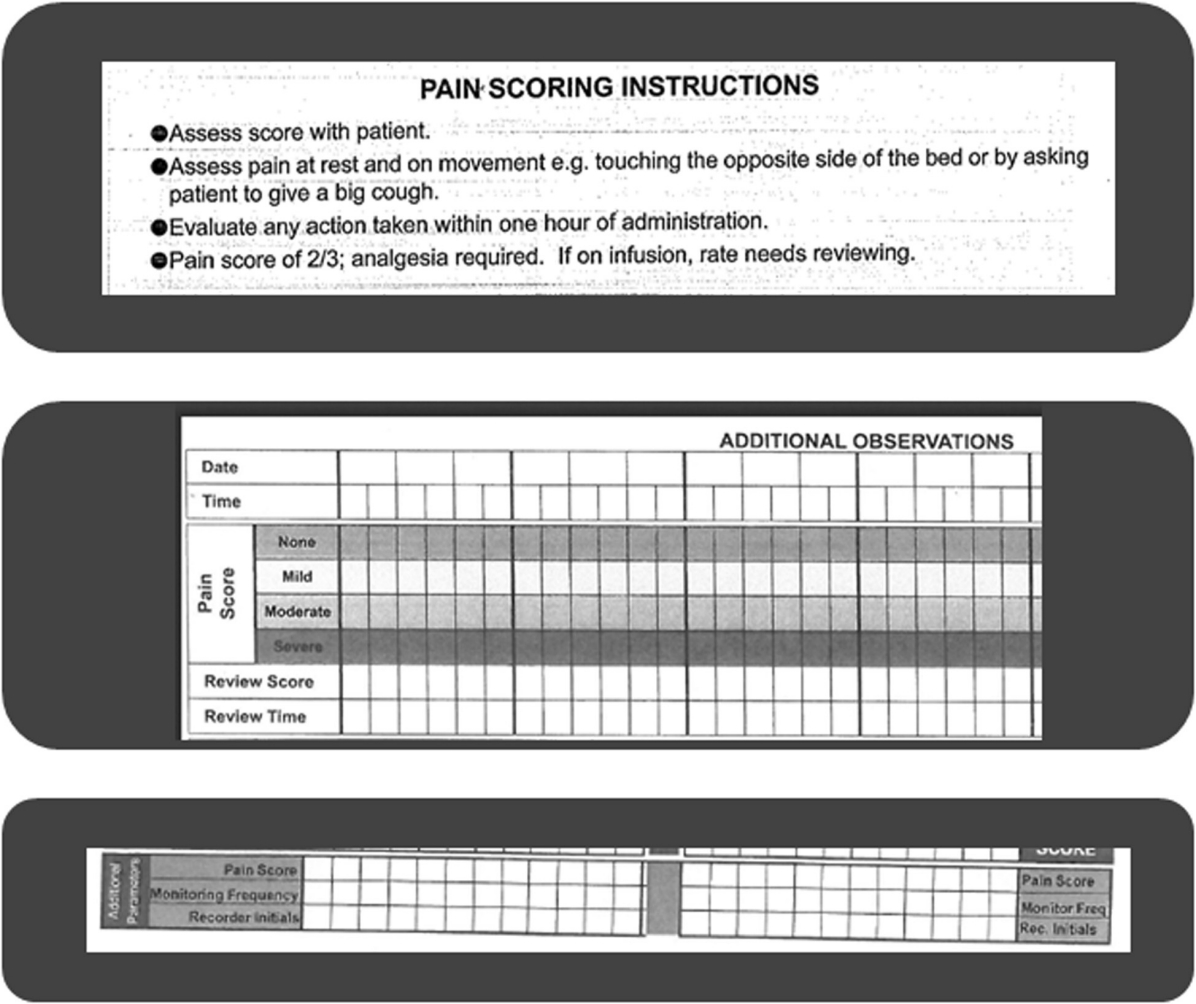
Examples of pain score entry sections and instructions (extracts from three systems in use in England). The figure presents extracts from Early Warning Scoring Systems including sections about the patient’s pain and related guidance

A variety of pain assessment and documentation tools were used across the different hospital settings, resulting in a patient’s pain being documented in a variety of different places. The tools were standardized within but not across hospitals. Among the tools available were, for example: acute pain assessment forms, with space to write a description of the pain experience and drawings of the human body to mark the location of pain; specialist nursing assessments documents, where chronic pain with mobility triggered further assessment, and acute pain with mobility triggered an analgesic ladder; or sections about pain were used within documentation of other assessment activities, such as wound care. In some of the wards, stocks of paper forms for documentation of clinical care activities included reference to the Abbey Pain Scale [30] or PACSLAC [31] - which are pain assessment tools designed for patients with dementia -, but these were not seen in use (nor indeed were easily available in the wards). None of the tools actually in use had been specifically designed to cater for the cognitive impairment of patients with dementia and the same tools were used with all patients regardless of their cognitive function. All four hospitals also used modified versions of the National Early Warning Score (EWS) chart [32] to record vital signs. Pain was an entry in these forms, with space for a score of pain intensity (Fig. 1).

In the hospital in Scotland a 0–10 scale was used with the EWS chart, with zero being no pain and 10 being the worst imaginable pain, while the three hospitals in England applied a scoring for pain on a 0-3 scale, with zero being no pain, and then 1, 2 and 3 for mild, moderate and severe pain. However, clinicians in England also reported using pain rating scales from 0–10 within other pain assessment charts. The hospital in Scotland and one of the hospitals in England also used a routine observation round (where health care assistants asked patients about their pain when doing comfort rounds) to identify the presence or absence of pain, documented with a Yes or No answer.

### The subjectivity of pain scores

Among our participants, there was a general sense that a 0–3 scoring range was simple and straightforward.

*… at least with zero to three, you can say, ‘Zero is none, one is mild, two is moderate, three is severe.’ Simple. [H4, nurse/deputy ward manager]*

However, some participants expressed the need for the increased granularity and “flexibility” provided by a 0–10 range.

More generally though there was awareness of the inherent subjectivity of the pain scores, and how pain for one person is not the same as pain for another. Scoring ‘sometimes felt a bit random’, with consequences for their usefulness for treatment decisions (clinical utility).

*I think one, nought to three, it doesn’t give you a great score of pain, and everybody's, it’s a subjective thing, isn’t it, it’s, pain, I mean, some people could stand on a nail and other people… [H1, staff nurse]*

*The scoring on that, sometimes it feels a bit random that they’re sitting having a cup of tea and a biscuit and they say oh, their pain is at nine and you think… in yourself, probably you’re thinking ‘it doesn’t… you don’t look as if… I wouldn’t have scored you at nine’ but it’s individual and we recognise that and if it is over say, above the four, then we have to go back to the patient. [H2, senior charge nurse]*

*…people really struggle with the numerical concept or scales or lines. We understand what we mean by it but your average person and certainly your average person with dementia, has no idea what you’re talking about if you say ‘oh 10 is the worst pain ever’ and, but even that’s not so helpful because it depends what they’re using as their reference worse pain ever. If they’ve only ever cut their finger before then they won’t have a reference point for, I don’t know, a burst appendix or something, so I’ve not found them [pain scales] desperately useful. [H1, medical consultant/elderly care]*

### Documenting the scoring activity, rather than the pain

Staff also shared with us concerns for the phenomenon of box-ticking, which they referred to as documenting simply to prove an activity has been carried out rather than for recording information for clinical care.

*…there’s the patient there – tick [H4, healthcare assistant/student nurse].*

*… [a pain assessment tool] has to add value, not just a tick box […] exercise. [H1, medical consultant]*

Our audits of patient records showed ambiguity in recording absence of pain, where the expression ‘no complaint of pain’, for example, was used to signify “*nothing to report*” [H1, nurse]. This, combined with the reported ‘need to tick the box’, suggests that a score of 0 may not necessarily correspond to a carefully assessed actual pain intensity but may just reflect that the nurse or healthcare assistant has ‘looked at’ the patient.

### Pain assessment for patients with dementia

Clinicians and support staff were aware that pain assessment tools and their related scoring systems could not be used in the same way with all patients, and especially with patients with severe dementia who would not be able to communicate their pain by using numbers. The patient’s body language and other cues would be studied to infer pain intensity and then a score entered on behalf of the patient, the staff having to make “some kind of judgment” on the basis of uncertain signs. Although there was no explicit complaint about these systems, we gathered a sense of awareness of the lack of accuracy of the associated process.

*And then again I think body language is a massive influence on how we would assess somebody’s pain who had dementia. But it will still be a scoring system that we would still use. […] With dementia patients, I think it’s quite difficult to use a pain score system if they’re not able to communicate with you. [H4, deputy sister]*

*Well I mean this is a very simple scale isn’t it? You know, none, mild, moderate or severe so it’s very, you know, it’s four words isn’t it? You have to try and fit it in to one of those categories. I mean it’s very basic…*

*Q: I’m thinking if a patient cannot tell you if it’s mild, moderate or severe.*

*Exactly, yeah and then you have to make some kind of judgement don’t you? So which is kind of one of those values - one of those arts and science things isn’t it? [H1, staff nurse]*

Only a physiotherapist was able to explain a method of associating apparent level of pain to a number in the 0–3 range on behalf of the patient. Her task was to help the elderly patients maintain mobility, and she would ask the patient to stand and move. In the physiotherapist’s eyes pain scores communicated information not on the intensity of pain but on the patient’s ability to mobilize despite the pain.

*…None [score = 0], is self-explanatory. Mild [score = 1], I would say is some pain but able to continue doing what I would want them to do. […] Moderate [score = 2] would be like impairing, you know, looking visibly distressed at doing what I’m asking them to do and severe [score = 3] I would say is they’re not able to do what I want them to do, that’s probably how I would grade it. […] I think that’s how I would, ‘cos it, I think you can be in pain but you can carry on, I would say that’s mild, they would be in mild pain, …. [H1, physiotherapist]*

As a consequence of the difficulty of recording pain scores on behalf of patients with cognitive impairment, these data were either not recorded, or used with caution. The level of trust in these numbers varied.

*…if a patient’s got dementia then it’s not really much use asking the patient what their pain scale [score] is. […] I don’t tend to go by it. [H1, doctor in training]*

*… personally I won’t put anything down when I’m filling it out unless he can tell me that he’s in pain or not […] because there’s no point trying to say yes, they are in pain, and then trying to say they are in severe pain because you don’t know [H1, support worker]*

*… well the thing is to try and enter something [a score] but it’s better, I mean it’s better not to say severe when it might not be severe …. [H1, support worker]*

It was explained to us that when the patient cannot communicate their pain in terms of numbers, pain assessment should be documented with the words ‘it appears that the pain is’ rather than the usual form ‘the pain is’; for example if a patient is not communicating the possibility of pain with their behaviour, documentation of this observation should be recorded as ‘it appears the patient is not in pain’ instead of the standard formula ‘no complaint of pain’. The equivalent score of 0 did not convey this nuanced difference, in either meaning or the source (patient or staff) of this data point.

*… unless they’re able to confirm it, if I said, “Oh, is it sore?”, and they said no, I’d put, “Appears to be in pain, but denies it when asked”. [H1, physiotherapist]*

### Pain scores and decision rules

During interviews clinicians indicated how they had both ‘formal’ and ‘informal’ decision rules (algorithms) associated with the scores on the pain assessment tool. In one case, for example, pain scores of 2 out of 3 triggered analgesia and then a repeated observation within 1 h (Fig. 1). In another setting a score of 3 out of 10 required observation every 2 h.

*There is a protocol, is somebody scores over three, you go back every couple of hours [H2, senior charge nurse]*

Other less explicit rules, which were apparent for all types of pain assessment tools included ‘common practice’ relating scores to associated types of analgesic drug, with reference to the pain ladder [33], and the timing of periodic observations.

*…if they say ‘one’, then they won’t need something like oramorph or oxycodone or something very strong. Probably need something like maybe codeine, paracetamol sometimes can just help. Or maybe codeine combined with the paracetamol, just add an extra level, that’s to keep it under control, you know. [H4, nurse deputy ward manager]*

It appeared that participants found scores useful if and when they thought the numbers triggered action (decisions) in a precise way. Thus a 0-3 range was seen as more precise in informing level of analgesic required than a 0–10 range, and therefore more useful. Indeed it might be said that the type of analgesic gave meaning to the number: 0 = no analgesia, 1 = paracetamol, 2 = mild opioids, 3 = stronger opioids, triggering intervention of the pain specialist team.

*I think the zero to three is much better than zero to ten. […] because people will tell you, ‘It’s a four,’ and you think, ‘What does a four mean?’ [what action corresponds to this number?] [H4, nurse deputy ward manager]*

However, given the subjectivity of pain scores, and especially when inferences on pain intensity had to be made on behalf of patients with cognitive impairment, clinicians used their own judgment in applying the pain ladder algorithm for administration of analgesics and support staff used judgement in applying the algorithm to alert a nurse (and a nurse to alert the doctor).

*I mean when do you alert somebody? It doesn’t say, moderate pain is that […]. What do you class as moderate pain? How can you, you know it’s, to me moderate pain is .. worse than a headache but less than, I don’t know, .. being in severe pain. But how do you? What’s moderate? How can you tell? [H1, support worker]*

The process of interpretation meant that these tools were considered ‘only as good as the person using it’ and that automatic triggers were (or had to be) replaced by what was referred to as ‘common sense’ – that is: the clinical knowledge and knowledge of the person used to assess each specific situation, ‘making connections’ across all sources of information, including the scores.

*…any tool is only as good as the person using it and common sense should… The nurse, the common sense of the nurse using it because you need to be able to adapt this to whatever situation you’ve got, not just stick to this as a formal [rule].. [H1, staff nurse]*

*I could assess a patient using my observation skills without looking at a score to know they were at a higher risk of something. So I think, in some way, we need to ensure that there’s professional judgement, there’s observation as well as a bit of calculation and prompt but certainly linking to other documentation and getting people to make these connections, to help them make the connections. [H2, nurse manager]*

### The temporality of pain assessment scores

By entering pain scores into the EWS charts at different times during the day, every day of a week, clinicians were able to visualize (more or less effectively, depending on the form design) trends and patterns of a patient’s pain. It was this information regarding the temporality of pain (rather than a score at a single point in time) that seemed to be most useful to the assessment of pain. Given that a patient’s pain often changes in time and fluctuates, a single point in time about presence/absence of pain or pain intensity was not representative of the patient’s problem. The process of tracking enabled clinicians perceiving trends and any changes to the usual state. It also enabled perceiving patterns, such as more pain in the morning and less at other times of the day.

*So each time you’re doing your observations, you’re documenting what the score is. So you’re keeping an eye. That way you can see is the pain more in the morning, more at lunchtime, … [H4, nurse deputy ward manager]*

*…you look at the pattern. You look at the numbers down here really. The final number. […] You look at the total. And you might see nought, nought, one, one, two, two, three, four, five and then you’d start looking across at this blood pressure, it might be going like that. […] you would look to why they had that number. [H1, staff nurse]*

However, a concern was raised in relation to how informative these data really were when considered in combination with the administration of analgesics. Only if pain assessment and documentation of pain scores were consistently done before or consistently after administration of analgesia, the visualization of the trend would inform on the efficacy of the therapy (whether ‘we are winning’, in the words of a medical consultant). Unfortunately the ‘logistics of the ward’ – that is the organizational routines of drug rounds and routines of observation rounds – meant these activities were often not synchronized and patients were assessed and data entered in the chart not consistently before (or after) medication.

*… but the times that they would do these, these are done on a morning after medication.[…] and on a night time, they’re done before the tablets. It’s just the logistics of the ward. It’s when we’ve got time to do ' em. So, you could have a, you could have an up and down thing like that, because one’s after tablets, one’s before. So you could have patients saying, “Yes, no, yes, no, yes, no”. [H1, staff nurse]*

## Discussion

In this paper we focus on the way in which pain assessment tools and their associated pain intensity scores are used to inform pain assessment and management, both in general and specifically for patients with dementia. Although on paper, and in the guidance on use, pain scores are ‘absolute’ numbers, usually on a 0-3 or 0-10 range, we found their meaning was relative; relative to the patient being assessed, relative to the healthcare professional using it, relative to the time it was recorded, and to the purpose for which it was recorded (e.g. to document ‘activity’, or document/communicate pain).

They were considered clinically useful, though not necessarily to inform decisions as designers intended. The clinical utility of documenting and keeping track of pain scores was twofold. First, the presence of thresholds (cut-off scores) triggered and guided actions. This we found was on three aspects of pain management: escalating care from HCAs to nurses and doctors; deciding on the level of medication required on the pain ladder [33]; and deciding the periodicity of subsequent observations (e.g. hourly or every two, four, six hours). Second, the process of tracking itself, by generating a series of data points in time, enabled the building of knowledge of the history of a patient’s condition and pattern recognition in terms of trends or sudden changes. Knowledge of the pattern, or most importantly of what was usual for the patient, appeared to be a prerequisite for deciding whether to act when the threshold was reached, and whether to respond to the pain score alert.

In contrast, a single pain score above a cut-off point was often not considered sufficient to elicit treatment, particularly in patients with dementia. The reasons resided in the subjective nature of patients’ pain reports and in staffs’ inevitably uncertain inferences of pain for patients with cognitive impairment. The scores were absolute numbers but their value was relative.

The varied level of confidence in the data had implications for whether or not pain assessment/documentation tools were used. Data on pain scores were not recorded and/or were used with caution. Likewise, staff indicated that the use of ‘decision rules’ to inform medication use, without consideration of the patient context was problematic. This is supported by a study carried out by the American College of Surgeons Committee on Trauma [34] which concluded that when a rule such that “all patients with a [score] of five or greater must be assessed” in practice means additional administration of opioids without regard for patients’ specific conditions, “that this may be fatal, especially in elderly patients.” [34]

### Implications for the design of CDSS for the assessment and management of pain in patients with dementia

It could be argued that because patients with dementia each present a different context, while CDSS depend on agreed and accepted rules, that use of these systems for this patient population is almost impossible. However, this concern is perhaps true for all patients, not only those with cognitive impairment; as any clinician would say: each patient is different, and providing a different context. Despite each patient being unique and pain being inevitably subjective, clinicians recognise the importance of measuring in some way a patient’s pain, as this is the way for them to know if ‘one is winning’, even if the measurement is imperfect and approximate. On the basis of these measurements some form of CDSS can be designed, as long as the recommendations the system produces (e.g. the wording of what action to take) and the ways it is implemented acknowledge the uncertainty inherent in the data and leave room for clinical judgement. In implementing CDSS, the emphasis should shift away from box-ticking or rule-following and towards support of professional judgement.

To know if ‘one is winning’ (or if the intervention has been effective), a single assessment in time is not sufficient; rather it is the history of the pain that is most informative. One of the issues with existing pain assessment tools is that they tend to record single assessment points, rather than trends or patterns in time. A CDSS would need to better integrate pain measurements with interventions over time to enable better understanding and monitoring of pain. To our knowledge, there are no existing tools that provide the basis for this at present.

The findings of our study support the view that pain scores have a clinical utility in terms of providing thresholds or cut-off points for action. It is expected that this would improve patient outcomes by alerting clinicians of the need for attention to the patient condition and informing decisions. However, a single score should not be used as an absolute measure of intensity of pain combined with a generalized algorithm of corresponding levels of medication, as this has potential serious, even lethal, consequences for patients. Instead, pain intensity cut-off points and alerting systems (triggers) should be personalized on a patient-by patient basis as suggested in earlier work [14, 16, 17]. How the personalisation of the alerting cut-off scores for individual patients with dementia may be done in practice should be explored in further research; it may involve some degrees of guidance from CDSS, and/or the same clinician tracking the patient’s pain over several days, making then a subjective decision about the cut-off point.

Given our results, it may be more appropriate to generate triggers or alerts on the basis of the trend and pattern generated for each patient by the repeated entries of data. For example when the trend is ‘steadily increasing pain’ or there are sudden variations beyond the usual for that patient, the alert should then be interpreted relative to the patient’s case history, and relative to the source of the data – being the patient’s self-report or staff inference.

Furthermore, as mentioned above, trends in pain scores are more meaningful when directly linked to timing of administration of analgesics. While this is difficult to achieve with paper based tools (such as those used currently in large number of hospitals in the UK), where administration is recorded in drug charts separate from observation charts, the linking of data should be more straightforward in electronic systems. Data entry may be done in separate screens but review of the data could be done on combined visualizations of the two on one screen. These visualizations could include graphic displays of the trends associated with pain scores, as well as indicating visually when/if analgesics and other pain relieving interventions had been administered.

An additional advantage of this type of computerized system could be for specialized teams – such as pain specialists – to screen records of inpatients so that they could visit and review those patients with documented pain not otherwise referred to them (similarly to what has been proposed for the screening of discomfort of cancer patients by palliative care teams [35]). This is also an example of the ‘new uses’ generated by the introduction of electronic systems in place of paper-based ones and how their clinical utility extends to new aspects of patient care.

The subjective nature of measures of pain intensity also has implications for data quality and for secondary use of these aggregate data across patients. Documentation of pain assessment through the use of a numerical score involves a loss of meaning, particularly of whether the source of the score was the patients themselves verbalizing the pain, or inference from members of staff, or family members. There is considerable literature to show that professionals scores and patients’ scores do not always correlate well. While the acronym APP (equivalent to ‘patient appears to be in pain’), for example, qualifies the documentation of appearance of pain in narrative form “when a pain intensity rating cannot be obtained” from the patient [36], this information is lost in numerical data entry and when all scores of the same number are treated the same.

There also needs to be caution when interpreting a zero score in documentation of a patient’s pain. A score of 0 may have been entered when with a quick look the patient did not appear to have pain but without a proper assessment; it would bear a meaning of ‘activity done’ rather than a sign of absence of pain. We recommend that in secondary use of the data for research or management purposes, users be especially wary of the meaning of *zeros* in pain score fields.

### Limitations

Although we collected data across a number of hospitals in the UK, using triangulation of methods, our results may not be transferable to other hospitals or health care systems; particularly where EHR systems (rather than paper documentation) are in use. Since most of the pain assessment tools designed for use with patients with dementia are observational (not involving asking patients to self-report), our study design did not include investigating the patients’ perspective on their experience of ‘scoring’ their pain; however, since we found that EWS, including scoring of pain, are used with patients with dementia, future research should include patients’ views on the use of these. Our findings about the clinical utility of pain scores emerged out of wider exploratory investigation of pain assessment and management practices; more focused research should be carried out specifically on the clinical utility of pain scores sections within EWS systems, especially in view of the computerization of these originally paper based forms into CDSS.

## Conclusion

When designing decision support tools, it is important to assess their potential clinical utility across a number of dimensions including their potential for clinical effectiveness, economic efficiency and usability and fit with organizational context and workflow. Our study suggests that for pain assessment and management, decision support interventions should personalize alerting cut-off scores for individual patients, and monitor and track pain scores over time, linking alerts and recommendations for action with trends in the data, rather than reacting to single individual data points. In patients with dementia, where staff are often inferring pain intensity from patient behaviour, rather than on the basis of patient reports of pain, further consideration needs to be given to supporting both documentation and treatment recommendations that recognize the uncertainty around such pain scores. There needs to be a recognition that whilst scores are absolute numbers, their actual meaning is relative, and flexibility should be built into any decision support system to recognize the essential role of professional judgment in both scoring and actions.

## Additional file

Additional file 1:
**Interview Guides.** Interview guides used for semi-structured interviews with staff and family members. (DOCX 205 kb)
