# Supplementary figures and images for: The relative meaning of absolute numbers: the case of pain intensity scores as decision support systems for pain management of patients with dementia
Source: BMC Med Inform Decis Mak. 2015 Dec 24;15:111. doi: 10.1186/s12911-015-0233-8 (PMC4690343; doi:10.1186/s12911-015-0233-8)

## Interview guide – patient’s family members (‘carers’)

| 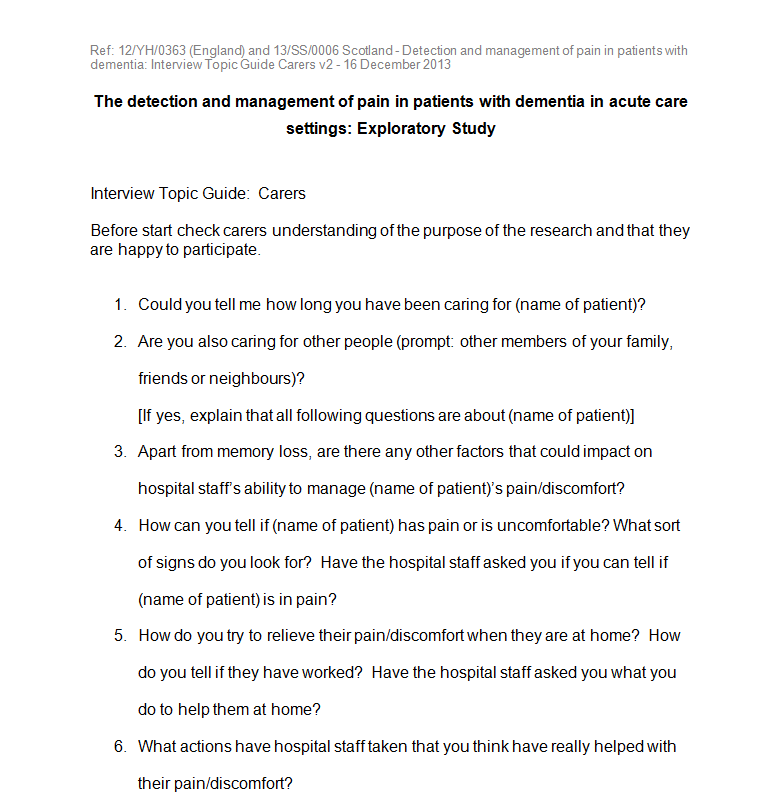 |
| --- |
| 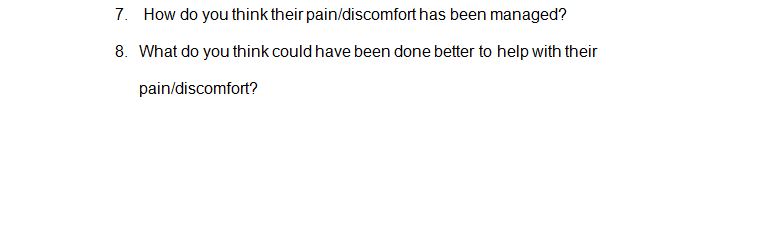 |
| 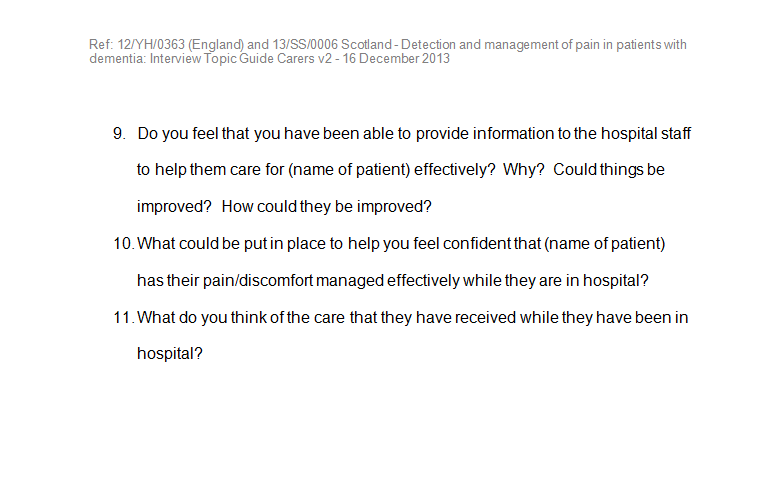 |
|  |

## Interview guide – ward staff

| 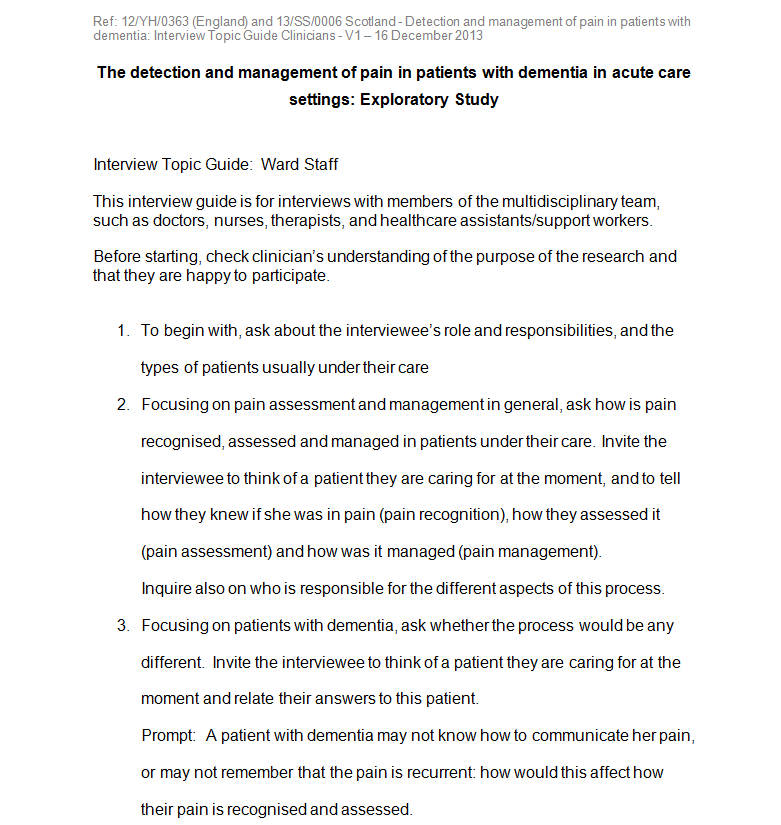 |
| --- |
| 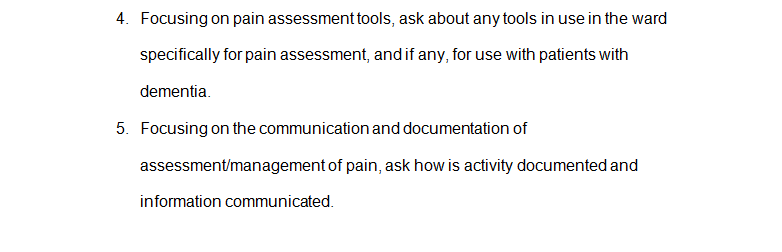 |
| 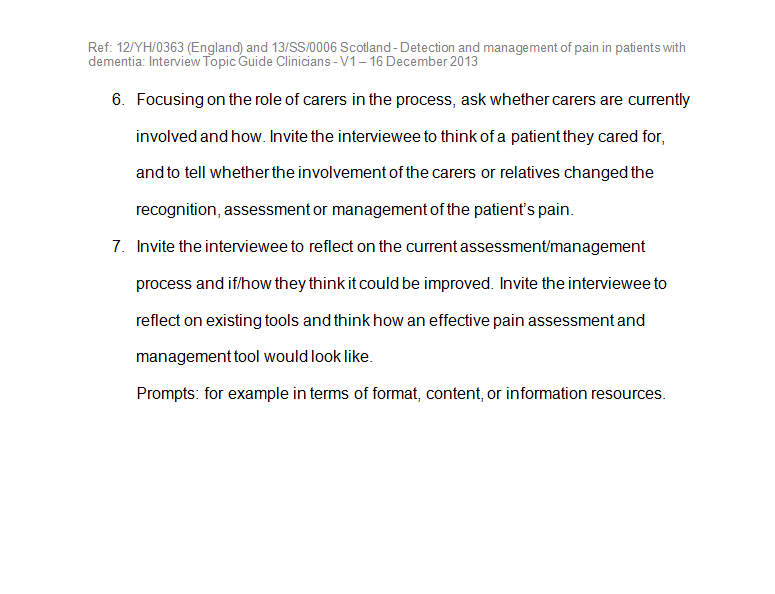 |
|  |
|  |

Supplement: Additional file 1: — Interview Guides. Interview guides used for semi-structured interviews with staff and family members. (DOCX 205 kb) [file 12911_2015_233_MOESM1_ESM.docx]
